# The Christmas Numbers

**Published:** 1892-12-24

**Authors:** 


### The Christmas Numbers.

Christmas numbers are as essentially part of our Christmas as—well, as plum pudding or mince pies, and, to our mind, being no longer in our teens, they are infinitely nicer. Their numbers grow greater every year, and to such an extent do they forstall Christmas nowadays that before long we shall expect to see on the bookstalls special summer and Christmas numbers combined. The competition in the literary matter, the illustrations, and covers they contain is very great, and novelty would be well nigh impossible were there not changing fashions in public taste. And so cleverly do the literary caterers understand their business that these Christmas numbers of our papers are really quite historical guides to the taste of their day. The illustrations within the pages are often most admirable, and the larger specimens of pictorial art, which turn our railway stations at the festive season into picture galleries, no doubt are pleasing to the many by reason of their popular subjects and vivid colouring. The brilliant complexions of the fairer sex, if cheering in the murky atmosphere of the "underground," are a little hard on nature as a rule. But that is all we have to complain of, and now let us turn our attention to the individual papers. Age must have priority, a fact which saves us from the difficulty of choice amongst so many pleasing subjects.

### THE ILLUSTRATED LONDON NEWS.

Our oldest and time-honoured friend, the *Illustrated London News*, contains a capital selection of stories and illustrations. There are none of those coloured productions in which the *Graphic* and other Christmas numbers indulge, but some of the prints are very good, notably one by Phil May of an inimitable modern young man of a certain stamp. This picture is, perhaps, the most modern in character of all the illustrations in the number, for there is a certain conservative tone about the *Illustrated* through which it runs very much in the same lines year after year in its "get up." Our old friend does not forget the youngsters, as most of the younger papers do, and this year the Christmas number commences with a fairy tale called "Princess Ice Heart." This is the history of a princess burdened with a stony heart by her fairy godmother. Every means are tried to make her tender hearted, and the king, her father, makes it known that any suitor who can draw tears from his daughter's eyes shall be her accepted husband. The result of each trial is failure, until at last a princely suitor conceives the idea of drawing tears from the princess's eyes through laughter. He accordingly becomes metamorphosed into an ape, and effects his object; the princess weeps with laughter, and the spell is broken. The usual happy ending follows. "Pallinghurst Barrow," by Grant Allen, the story which follows, illustrates the terrible effect the recitation of a gruesome legend may have upon excited nerves, and the horrible experiences of the hero in consequence should be a warning to tale-tellers and hearers alike. The story is illustrated by weird and uncanny pictures, which are liable to produce those nice "creepy" feelings which are part and parcel of a proper Christmastide. "The Rev. Ezekiel Crump," by Frank Stockton, is the next item on the programme. "The Rev. Ezekiel Crump" was a pelargonium, though he doesn't sound like it. The care of him leads the florist, who would exhibit him at a show into some amusing adventures. In the course of these he unwillingly overhears a proposal whilst occupying a cistern, much against his will. "The Rev. Ezekiel Crump" ends by coming into the possession of the heroine of the proposal scene, and does not go to the show. "The Culminating Point" will be welcomed by the boys, and "The White Hat," an amusing little medley, closes the volume. In reading this last sketch we were a little distracted by Sir John Bennett, Aspinall's Enamel, Torpid Liver, and Maple and Co. A perusal of the *Illustrated London News* will explain to our readers how this happened if they are inclined to doubt our sanity!

### THE GRAPHIC.

Next comes the *Graphic*. This year's number is admirable in many ways, and if not a very bulky volume, all that is within its cover is excellent. The literary portion is certainly not large, but of delightful illustrations we have our fill. The young ones will think this a charming number by reason of its many pictures, and the older folk must find something to please in the stories it contains, though their number is not large. "Two of Them," the opening story, is a clever little modern sketch. There are only "two of them," a man and a girl. They are friends, and being much impressed with the charms of platonic friendship, they enter into a bargain that "he" shall treat "her" like a man—like his friend Thomson in fact. The bargain gives rise to a good deal of amusing confusion, and it is not quite fairly carried out by one of the parties. Mary, the heroine, honestly tries to follow in Thomson's footsteps; but the hero has not a conscience, and Thomson would have been much surprised at the "ways" put down to his charge. The end of the bargain is all our fair readers could wish, and though only told by inference, is very unmistakable. The sketch is nearly entirely dialogue throughout, and very amusing dialogue too. After this follows a pictorial story, in pretty "Caldecott" tints. Very widely different is "Ivan Greet's Masterpiece," the tale which follows. Opening with a convivial scene, it ends a hopeless tragedy. Mr. Grant in his story has, so to speak, "burnt his ships behind him," and literally nothing remains at the end but ashes. The young author, hero of the tale, bright with hope, seeks the shores of Jamaica, buys his two acres of land, builds himself a hut, and settles down to life amongst the negroes, thinking away from civilisation he will secure quiet times for inspiration. His philosophical poetic nature revels in the simple, almost savage, life around him, and he writes under this influence a book embodying his experiences in these new scenes. This is completed, and satisfies even his critical judgment. He is about to despatch it for publication in Europe, when he is stricken down with fever, and dies. The romance of the story is concentrated in the devotion of a half-cast girl. Resolved that his wishes as to his book shall be carried out, although he exists no more, she sets herself the task of earning the necessary price for its publication. She keeps the precious manuscript carefully stored in her hut, whilst working steadily towards her end. In her absence one day the hut is burnt down, and with it perishes the precious volume. Overcome by the tragedy, she sinks by the smouldering remains of her dwelling, and, after a night of tempest, the negroes find her dead in the morning. And "thus it is," as is written in the conclusion of the story, "that the world has never heard more of 'Ivan Greet's Masterpiece.'" The last literary item on the *Graphic* programme is "Owen Wingrave," a ghost story, by Henry James. The mystery is not explained, and rats are left out of the question! Having said so much, we will not spoil the zest of the reader by betraying the secrets of the story, which is sure to charm those who revel in the gruesome delights of a ghost story.

### THE LADY'S PICTORIAL.

The *Lady's Pictorial*, quite one of our most youthful friends, contains but one story, but as that one is full of striking incident and most powerfully illustrated by Maurice Greiffenhagen, we do not think its readers will complain. The plot of "A Wild Proxy" is certainly a wild conception, and were it not that Mrs. Clifford knows well how to tell her story, there would not be a sufficient element of probability in it to sustain our interest. Two very nice ordinary people become engaged to one another, and get as far as the marriage ceremony and Charing Cross afterwards, when the wild element in the shape of a cousin of the bridegroom steps in and alters the whole ordinary sequence of affairs. Previous to the hero, Lawrence Halstead's, engagement, this wild cousin falls somewhat in love with the heroine, Helen Lambert. Following the proverb that "forbidden fruit is sweet," his love is not lessened when the engagement takes place. Subsequently he acts as best man at the wedding, and part of his duties being the travelling arrangements of the honeymoon, he undertakes to despatch the young couple from Charing Cross. Very late for the train they arrive. There is an accident in connection with the carriage that brings them. The bridegroom steps out to see that due assistance is rendered to the sufferer from the mishap, and leaves the bride in charge of Frank Merreday, this cousin. A wild project enters Frank's head. He hurries Helen to the train, gets in himself, and they are borne out of the station. He passifies Helen in her astonishment and alarm by plausible explanations, and the prospect of a speedy meeting with her bridegroom at Dover. But Dover does not end the separation, and by a series of telegrams despatched apparently by Lawrence Halstead to him, but in reality directed by Frank to himself, he lures the most unwilling, perplexed, and miserable bride right away to Genoa. Her unhappiness causes him at last to confess to his deeds, and then, of course, all the complications of such a situation rush in. In the conclusion the story returns to ordinary channels, but many difficulties and varied scenes are presented to the reader before this takes place. The sad end of the poor "Wild Proxy" casts a halo of romance over his unsatisfactory life, and makes the reader accord the forgiveness he receives from the actors in the story.

